# Integrated bioinformatics and experimental validation identifies SOX2 as a key therapeutic target for chronic atrophic gastritis/gastric cancer-depression comorbidity

**DOI:** 10.3389/fonc.2026.1858473

**Published:** 2026-08-31

**Authors:** Yang Liu, Yuefen Wang, Cuimei Zhao, Wenbo Li, Zongmin Ma, Chen Wang, Zhongxia Yuan, Shaomin Wang, Nan Zhang, Yangang Wang

**Affiliations:** 1 Hebei University of Chinese Medicine, Shijiazhuang, Hebei, China; 2 Beijing Hospital of Traditional Chinese Medicine, Capital Medical University, Beijing, China; 3 Jiaozhou Hospital of Traditional Chinese Medicine, Jiaozhou, Shandong, China; 4 The Fourth Affiliated Hospital of Hebei University of Chinese Medicine, Shijiazhuang, Hebei, China; 5 Beijing University of Chinese Medicine Third Affiliated Hospital, Beijing, China; 6 Hebei Province Hospital of Chinese Medicine, Shijiazhuang, Hebei, China

**Keywords:** chronic atrophic gastritis, depression, gastric cancer, inflammatory mechanism, SOX2, stomach-brain axis

## Abstract

**Objective:**

Gastric cancer (GC) and its precursor chronic atrophic gastritis (CAG) exhibit a high comorbidity with depression. However, the core molecular mechanisms underlying stomach-brain axis-mediated crosstalk between GC/CAG and depression remain poorly clarified. This study attempted to screen pivotal molecules correlated with the bidirectional correlation between GC/CAG and depression, and explore the inflammatory regulatory network through integrated bioinformatics analysis and systematic *in vivo* and *in vitro* validation, so as to provide potential clues for revealing their interactive relationship.

**Methods:**

We integrated transcriptomic datasets of CAG, GC, and depression to identify shared differentially expressed genes, further prioritized core diagnostic genes by machine learning, and characterized their biological functions through functional enrichment analysis. *In vivo* validation was performed in MNNG-induced CAG/GC rat models, including behavioral tests, histopathological evaluation, and gene expression analysis of gastric and brain tissues. Molecular docking and molecular dynamics simulations were performed to assess the binding affinity and structural stability between MNNG and SOX2. *In vitro* experiments explored how SOX2 expression levels correlate with NOTCH signaling/IL-6 in gastric epithelial cells and paracrine effects on neuronal inflammation.

**Results:**

A total of 21 overlapping differentially expressed genes were screened out, among which 8 core diagnostic genes with AUC values greater than 0.7 were identified, and SOX2 presented the highest diagnostic efficiency with an AUC of 0.849. The MNNG-induced model rats displayed reduced activity and anhedonia-like behavior, accompanied by decreased SOX2 expression and activated NOTCH/IL-6 signaling in gastric and brain tissues. Molecular docking results confirmed that the binding energy between MNNG and SOX2 was −5.5 kcal/mol. *In vitro* cellular assays revealed an association between SOX2 downregulation and NOTCH cascade activation alongside elevated IL-6 secretion; neuronal cells cultured with gastric cancer cell-derived conditioned medium exhibited concurrent IL-6-related inflammatory alterations.

**Conclusion:**

This study characterizes the bidirectional correlative relationship between GC/CAG and depression mediated by the stomach-brain axis. SOX2 is characterized as a key molecule correlated with the regulation of the NOTCH/IL-6 signaling pathway in this interactive network, indicating its potential as a diagnostic biomarker and therapeutic target for GC/CAG complicated with depression.

## 1 Introduction

Gastric cancer (GC) is the fifth most common malignant tumor and the third leading cause of cancer-related death worldwide (1, 2). Its carcinogenic process follows the classic Correa cascade, progressing from chronic non-atrophic gastritis to chronic atrophic gastritis (CAG), intestinal metaplasia, dysplasia, and ultimately invasive GC (3, 4). Clinically, patients with CAG and GC are frequently accompanied by psychological comorbidities, among which depression has the highest prevalence, reaching up to 58.06% in GC patients, significantly higher than that in the general population and other digestive system malignancies (5, 6).

The stomach-brain axis constitutes a bidirectional communication system between the stomach and the central nervous system (CNS). It sustains systemic homeostasis via neural, immune, endocrine, and metabolic pathways, serving as the fundamental structural basis for the interplay between gastric pathological lesions and mental disorders (7, 8). Cumulative evidence indicates that pro-inflammatory cytokines derived from damaged gastric mucosa can enter the systemic circulation, traverse the blood–brain barrier, trigger microglial activation and central neuroinflammation, and eventually correlate with the emergence of depressive-like phenotypes (9). Nevertheless, the core shared regulatory genes that coordinately modulate gastric mucosal injury and central neuroinflammation during GC-depression comorbidity remain poorly defined, representing a critical research gap in this field.

In recent years, integrated bioinformatics analysis combined with machine learning algorithms has become a powerful tool for deciphering the molecular mechanisms of complex comorbid diseases and screening core regulatory targets (10). SRY-box transcription factor 2 (SOX2), a core member of the SOX gene family, is a key regulator of tissue homeostasis, cell differentiation, and inflammatory response (11). Accumulating evidence has validated that SOX2 silencing induced by promoter hypermethylation is associated with the malignant progression of GC by suppressing gastric epithelial cell differentiation and apoptosis (12) (13). Mechanistically, the highly conserved NOTCH signaling pathway acts as a central downstream effector driving gastric inflammatory deterioration and carcinogenesis. Persistent aberrant activation of NOTCH signaling is commonly observed in CAG and early-stage GC lesions (14). Dysregulated NOTCH signaling disrupts gastric epithelial homeostasis, correlates with aberrant cell proliferation and inflammatory infiltration, and is associated with the sequential progression of gastric atrophy, intestinal metaplasia, and malignant transformation (15). Moreover, activated NOTCH signaling functions as a crucial upstream initiator of the IL-6-mediated inflammatory cascade, establishing a vital link between peripheral gastric inflammation and central neuroinflammation. Neurobiological studies have further confirmed that NOTCH activation substantially upregulates the transcription and secretion of pro-inflammatory IL-6 (16). Circulating peripheral IL-6 penetrates the blood–brain barrier, activates microglia, and provokes sustained neuroinflammatory responses, thereby promoting the onset and exacerbation of depressive phenotypes (17). Importantly, previous mechanistic studies have established a definitive negative regulatory relationship between SOX2 and the NOTCH pathway in multiple digestive and inflammatory models. Downregulation of SOX2 abolishes its inhibitory effect on NOTCH signaling, leading to excessive activation of the NOTCH cascade and subsequent pro-inflammatory responses (18, 19).

Therefore, this study hypothesized that SOX2 downregulation may be associated with the bidirectional crosstalk between GC/CAG and depression via the stomach-brain axis: reduced SOX2 abundance in gastric mucosa correlates with NOTCH signaling activation and IL-6-mediated inflammatory response during CAG/GC progression; peripheral inflammatory cytokines enter the systemic circulation and correlate with central neuroinflammation and depressive-like phenotypes; meanwhile, SOX2 downregulation in the CNS further correlates with aggravated neuroinflammation, forming a vicious correlative cycle between gastric lesions and depression. In this study, we integrated transcriptomic bioinformatics, machine learning, *in vivo* animal models, and *in vitro* cellular assays to verify this hypothesis, aiming to identify the core regulatory target of GC-depression comorbidity and provide a novel theoretical basis for its clinical management.

## 2 Methods

### 2.1 Acquisition of disease-related transcriptomic data and candidate genes

Four transcriptomic datasets covering both CAG and GC lesions (GSE118916, GSE54129, GSE65801, GSE60427) were retrieved from the Gene Expression Omnibus (GEO) database hosted by the NCBI. The raw data of each dataset was preprocessed using the RMA algorithm for background correction and normalization, and batch effect correction was performed using the ComBat function of the sva package in R. Differentially expressed genes (DEGs) between GC tissues and adjacent non-tumor tissues were screened using the limma package, with the threshold set at |log2 fold change (FC)| ≥ 1 and adjusted P-value < 0.05.

Depression-associated candidate genes were systematically curated from three authoritative databases: the Alliance of Genome Resources Database, GeneCards (relevance score ≥ 1.0), and UniProt (reviewed entries only). Subsequently, intersection analysis was performed between CAG/GC-related DEGs and depression-associated candidate genes, and the identified overlapping genes were subjected to subsequent functional and mechanistic analyses.

### 2.2 Weighted gene co-expression network analysis (WGCNA)

WGCNA was performed using the WGCNA package in R to identify CAG/GC-related gene co-expression modules. The optimal soft-thresholding power was determined to construct a scale-free co-expression network (scale-free R² ≥ 0.85). Module-trait correlation analysis was performed to evaluate the correlation between each module and CAG/GC pathological phenotype, and the module with the highest correlation coefficient with CAG/GC was selected as the key phenotype-related module. Genes in the key module were intersected with CAG/GC DEGs to obtain CAG/GC-associated candidate genes for subsequent analysis.

### 2.3 Functional enrichment analysis

Gene Ontology (GO) and Kyoto Encyclopedia of Genes and Genomes (KEGG) pathway enrichment analyses of the overlapping genes were performed using the clusterProfiler package in R, with a statistical threshold set at adjusted P-value < 0.05. The results were visualized as dot plots using the ggplot2 package.

### 2.4 Screening of core genes based on stacked ensemble machine learning

Based on the gene expression profile data of CAG/GC datasets, the samples were randomly divided into a training set (70%) and an internal validation set (30%) using stratified sampling to maintain consistent class distribution. Four classic machine learning algorithms, including Least Absolute Shrinkage and Selection Operator (LASSO), Random Forest (RF), Support Vector Machine-Recursive Feature Elimination (SVM-RFE), and Extreme Gradient Boosting (XGBoost), were used to construct diagnostic prediction models for GC. Hyperparameter optimization was performed via 5-fold cross-validation. The performance of each model was comprehensively evaluated based on the Area Under the Receiver Operating Characteristic Curve (AUC), accuracy, and F1 score. A stacked ensemble learning strategy was adopted to integrate the prediction results of the four individual models, and genes with the highest feature importance and diagnostic AUC were identified as core diagnostic genes.

### 2.5 Molecular docking and molecular dynamics (MD) simulation

The 2D structure of N-Methyl-N’-nitro-N-nitrosoguanidine (MNNG, CID: 7604) was retrieved from the PubChem database (https://pubchem.ncbi.nlm.nih.gov/, accessed December 22, 2025) (20), and the 3D crystal structure of human SOX2 (PDB ID: 1O4X) was obtained from the RCSB Protein Data Bank (PDB, https://www.rcsb.org/, accessed on December 22, 2025) (21). The SOX2 protein structure was preprocessed by removing water molecules and non-specific ligands, adding hydrogen atoms, and repairing missing amino acid residues using PyMOL 2.5 software.

Molecular docking was performed using the CB-Dock2 server (http://183.56.231.194:8001/cb-dock2/index.php, accessed December 22, 2025), which integrates cavity detection, blind docking and homologous template fitting for protein-ligand interaction prediction (22). The docking parameters were set as default, and the conformation with the lowest binding energy was selected for subsequent MD simulation. MD simulations were conducted using Gromacs 2025 software (23). RESP charges of the MNNG ligand were calculated using ORCA 5.0 (24, 25). The protein was parameterized with the AMBER99SB-ILDN force field, whereas ligand parameters were derived from the General Amber Force Field (26). The protein-ligand complex was centered in a cubic simulation box with a minimum distance of 1.0 nm from the box edges and solvated with TIP3P water molecules (27). The system charge was neutralized prior to energy minimization. Subsequently, a two-step equilibration was performed: first under the NVT ensemble and then under the NPT ensemble, each lasting 200 ps with a 2 fs time step and positional restraints imposed (28). Finally, unrestrained production simulations were run for 100 ns. Topological and coordinate files were processed using Multiwfn (version 2.49.0), and parallel computations were enabled through the Message Passing Interface (29).

### 2.6 Drugs and reagents

MNNG was purchased from Macklin Biochemical Co., Ltd. (Shanghai, China), with the batch number of M813171. Sodium salicylate was obtained from Aladdin Biochemical Technology Co., Ltd. (Shanghai, China), and its batch number was F2124136.

### 2.7 Animals

Male Sprague-Dawley (SD) rats (body weight 150–180 g, aged 2 months) were procured from Sibeifu (Beijing) Biotechnology Co., Ltd. (Laboratory Animal Use License No. SYXK[Ji]2023-012, Beijing, China). Exclusively male SD rats were utilized in the present study. The rats were housed under controlled environmental conditions: temperature maintained at 24 ± 4 °C, relative humidity at 50%–70%, an internal-external pressure difference of ≥ 10 Pa, and a consistent 12-hour light–dark cycle implemented daily. This study was conducted in strict compliance with the guidelines and regulations formulated by the Laboratory Animal Welfare and Ethics Committee of Hebei Provincial Hospital of Traditional Chinese Medicine, which granted ethical approval (No.IACUC-HPHCM-2025-010). All experimental procedures were performed in adherence to the 3R principles for the ethical use of laboratory animals. Based on our team’s mature modeling protocol and published literatures, we constructed a progressive gastric lesion model in rats by long-term free drinking of MNNG solution (200 μg/mL) combined with intermittent fasting and sodium salicylate intervention. All rats belonged to a single unified animal cohort rather than independently raised batches. Modeling procedures were initiated in all model rats at 2 months of age. To characterize the sequential pathological progression from CAG to GC, all model animals from the same initial cohort were randomly allocated to two endpoints and sacrificed at distinct disease stages: one subgroup received 4 months of modeling treatment and was harvested as the CAG cohort, while the other underwent 8 months of modeling and was collected as the GC cohort. For statistical comparisons, the 4-month modeling CAG rats were paired with age-matched 6-month-old normal control rats under identical housing conditions, and the 8-month modeling GC rats were matched against 10-month-old normal control rats with the same feeding regimen. During the whole modeling period, the model group received an alternate fasting-feeding cycle (1 day of complete fasting followed by 1 day of ad libitum access to standard rodent chow). On every fasting day (once every two days), a single intragastric administration of 2% sodium salicylate solution was performed in the morning at a fixed volume of 10 mL/kg body weight; no drug intervention was conducted on non-fasting days. Rats in the age-matched control groups were provided with free access to standard pellet feed and clean drinking water throughout the whole experiment without any fasting treatment or sodium salicylate gavage (8, 30, 31).

### 2.8 Sample collection

Rats were fasted overnight and euthanized via isoflurane inhalation at 4 and 8 months after modeling initiation, respectively. Briefly, anesthesia was induced with 3–5% isoflurane (Cat. No. R510-22-2, RWD Life Science Co., Ltd., China) in oxygen using an anesthesia chamber, and maintained at 1.5–2.5% until the complete loss of pain-related reflexes was confirmed. Under aseptic conditions, abdominal aortic blood was collected first; whole blood samples were centrifuged to obtain serum or plasma, which was aliquoted and stored at −80 °C for subsequent detection. After blood collection, isoflurane administration was continued until complete respiratory and cardiac arrest was verified. Gastric and brain tissues were then harvested aseptically, with a portion of each tissue fixed in 4% paraformaldehyde for histopathological and immunohistochemical analyses, and the remainder snap-frozen in liquid nitrogen and stored at −80 °C for downstream molecular assays. All procedures complied with institutional animal welfare guidelines and the 3R principles to minimize animal distress.

### 2.9 Open-field test (OFT)

The OFT quantified general locomotor and exploratory activity as a readout of global motor function, conducted as previously described (32). The test was conducted in a square open-field arena (100 cm × 100 cm × 40 cm, RWD Life Science Co., Ltd., China) under uniform bright illumination. Each rat was gently placed in the center of the arena, and its locomotor activity was recorded for 5 min by an automated video tracking system. The total distance traveled was quantified as the primary outcome. Between trials, the arena was thoroughly cleaned and disinfected with 75% ethanol to eliminate residual olfactory cues (33).

### 2.10 Elevated plus maze (EPM)

The EPM evaluated anxiety-related avoidance; open-arm duration was the primary metric for anxiety-like traits. The apparatus (RWD Life Science) contained two open and two closed arms (10 cm × 50 cm each). Rats were placed on the central platform facing an open arm and explored freely for 5 min. Arm entry was recorded when all four paws stepped into the arm, and residence time stopped once the rat returned to the center. The maze was cleaned with 75% ethanol between subjects to eliminate odor cues (34).

### 2.11 Sucrose preference test (SPT)

The SPT quantified anhedonia-like depressive phenotypes as previously reported (35). Studies have shown that the 24-h sucrose preference test yields more stable data (36). The SPT consisted of a 48-h habituation phase followed by a 24-h formal test. During the first 24 h of habituation, rats were housed individually with two bottles of 1% (w/v) sucrose solution to acclimate to sweet taste (37). In the subsequent 24-h dual-bottle adaptation period, rats were provided with one bottle of 1% sucrose solution and one bottle of tap water to establish a two-fluid selection habit. After the 48-h habituation, a 24-h formal test was conducted, in which individually housed rats had free access to two pre-weighed bottles filled with 1% sucrose solution and tap water, respectively (34). To eliminate experimental bias induced by side preference, the positions of the two bottles were swapped every 12 h. Fluid consumption was determined by measuring the weight difference of each bottle before and after the test. Sucrose preference percentage was calculated as the ratio of sucrose consumption to total fluid consumption, with the formula as follows: Sucrose preference (%) = sucrose intake/total fluid intake × 100. To eliminate confounding effects induced by intergroup differences in rat body weight, total fluid intake was normalized and expressed as grams per 100 grams of body weight, calculated by the equation: Corrected total fluid intake (g/100 g BW) = (total fluid consumption/body weight) × 100.

### 2.12 H&E, AB-PAS, immunohistochemical (IHC), and immunofluorescence (IF) staining

Gastric and brain tissues from rats were fixed in 4% paraformaldehyde at 4 °C for 24–48 hours, followed by dehydration through a graded ethanol series, clearing in xylene, embedding in paraffin wax, and serial sectioning at a thickness of 4 μm. Prior to IHC and IF staining, paraffin sections were deparaffinized, rehydrated, and subjected to heat-mediated antigen retrieval to restore antigenicity. For gastric tissue sections, hematoxylin and eosin (H&E) staining was performed for histological assessment, Alcian blue-periodic acid-Schiff (AB-PAS) staining was conducted to visualize mucin-secreting cells and glycoconjugates, and both IHC and IF staining were applied to evaluate the localization and expression levels of target antigens. For brain tissue sections, only H&E, IHC and IF staining were performed, with AB-PAS staining omitted.

For cell samples, including gastric epithelial cell lines and PC12 cells treated with gastric epithelial cell-derived conditioned medium (CM), cells were seeded on sterile glass coverslips, cultured to appropriate confluence, fixed with 4% paraformaldehyde for 15 min at room temperature, permeabilized with 0.5% Triton X-100, and blocked with 5% bovine serum albumin to eliminate non-specific binding. Coverslips were then incubated with primary antibodies against target proteins overnight at 4 °C, followed by incubation with fluorophore-conjugated secondary antibodies for 1 h at room temperature in the dark. Nuclei were counterstained with DAPI, and coverslips were mounted with anti-fade mounting medium for subsequent fluorescence imaging.

### 2.13 Western blot (WB) analysis

Gastric and brain tissues were lysed in RIPA Lysis Buffer (ATYJ4161, Abbkine, China) supplemented with Protease Inhibitor (P1045-1, Beyotime, China) and Phosphatase Inhibitor (P1045-2, Beyotime, China). The protein lysates were separated by SDS-PAGE and subsequently transferred onto nitrocellulose membranes via electroblotting. The membranes were blocked at room temperature for 30 minutes using a Protein-Free Rapid Blocking Buffer (G2052, Servicebio, China) and then incubated overnight at 4 °C with the respective primary antibodies. Following primary antibody incubation, the membranes were probed with horseradish peroxidase (HRP)-conjugated secondary antibodies for 1 h at room temperature. Target protein bands were visualized and quantified using a gel imaging analysis system.

### 2.14 Conditioned medium (CM) preparation and cell treatment

GES-1, AGS, and MC cells were cultured in serum-free medium for 24 h upon reaching 80% confluence. The supernatant was collected and centrifuged at 1000 rpm for 10 min to remove cell debris, and the resulting supernatant was used as conditioned medium. PC12 cells were incubated with a 1:1 mixture of conditioned medium and complete culture medium for 24 h, after which the cells were harvested for subsequent assays.

### 2.15 Statistical analysis

All quantitative data are presented as the mean ± standard error of the mean (SEM). All statistical analyses were performed using GraphPad Prism 10.1 (GraphPad Software, San Diego, CA, USA). Prior to statistical evaluation, the Shapiro–Wilk test and Brown–Forsythe test were applied to verify data normality and homogeneity of variance, respectively. Only data satisfying both normal distribution and homoscedasticity were subjected to parametric statistical analyses. Specifically, unpaired Student’s t-tests were used for comparisons between two independent groups, whereas one-way analysis of variance (ANOVA) followed by Tukey’s *post hoc* multiple comparison test was employed for analyses involving three or more experimental groups.

All sample sizes for *in vitro* cellular and *in vivo* animal experiments were prospectively determined via statistical power analysis to ensure sufficient statistical reliability, with a two-sided significance level (α) of 0.05 and a statistical power (1−β) of 0.80 based on preliminary experimental results and published evidence. To adapt to different degrees of biological variability, we adopted differentiated biological replicates: n = 3 per group for stable cellular and molecular assays, and n = 6 per group for *in vivo* animal experiments with inherent inter-individual variation. Power verification confirmed that these sample sizes were adequate to identify statistically and biologically significant differences while minimizing false-positive and false-negative results. No samples or data were excluded during analysis, and all statistical comparisons were performed based on the original experimental grouping. Given the multiple phenotypic, molecular, and longitudinal endpoints evaluated in this study, Bonferroni correction was applied to control the family-wise Type I error rate and eliminate false-positive inflation caused by multiple comparisons.

## 3 Results

### 3.1 Data preprocessing and batch effect correction

**Figure 1:**
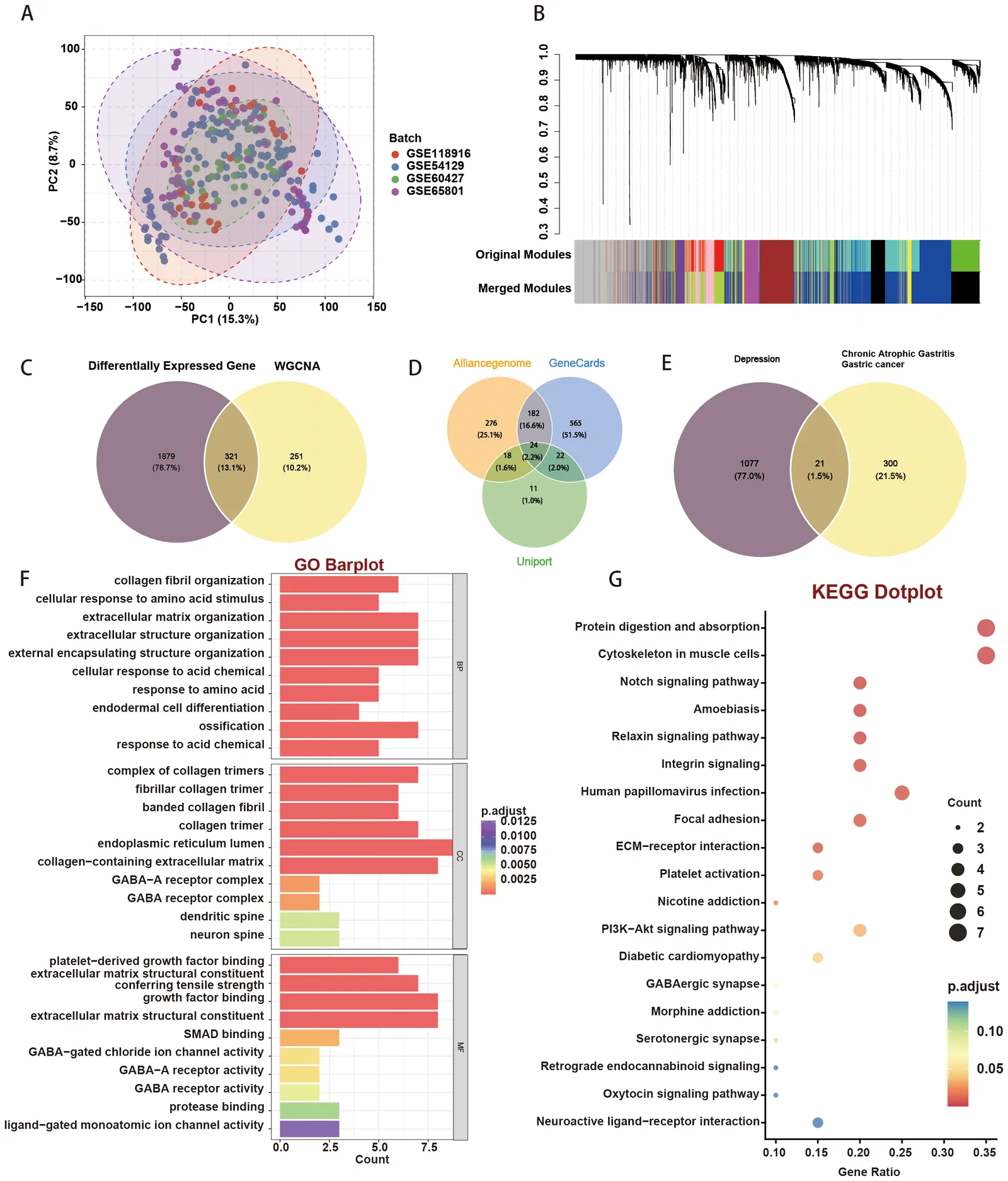
Shared molecular signatures underlying the crosstalk among depression, chronic atrophic gastritis and gastric cancer. **(A)** PCA scatter plot post batch-effect correction demonstrates successful integration of four datasets (GSE118916, GSE54129, GSE60427, GSE65801), with markedly mitigated batch heterogeneity. **(B)** WGCNA-derived gene dendrogram presenting hierarchical clustering based on gene co-expression patterns. **(C)** Venn diagram quantifying intersections between differentially expressed genes and WGCNA module genes. **(D)** Venn diagram showing overlaps of depression-associated genes curated from Alliancegenome, GeneCards and UniProt databases, which collectively constitute the full depression-related gene pool. **(E)** Venn diagram showing overlapping gene sets among depression, chronic atrophic gastritis, and gastric cancer. **(F)** Gene Ontology (GO) enrichment dot plot displaying top terms in biological process (BP), cellular component (CC), and molecular function (MF). **(G)** KEGG pathway enrichment dotplot.

Principal Component Analysis (PCA) confirmed that batch effect correction successfully harmonized the integrated transcriptomic datasets. After correction, samples from different biological groups exhibited more cohesive clustering patterns, validating the effectiveness of data integration (**Figure 1A**).

### 3.2 Identification of differentially expressed genes and WGCNA co-expression modules

For WGCNA, the optimal soft-thresholding power was determined to construct a scale-free co-expression network, and distinct co-expression modules were identified based on hierarchical clustering (**Figure 1B**). By integrating differentially expressed genes and WGCNA module genes, we identified 321 gastric cancer-associated genes (**Figure 1C**). Meanwhile, we curated 1098 depression-related genes by compiling candidate genes from multiple authoritative databases including AllianceGenome, GeneCards, and UniProt (**Figure 1D**). Intersection analysis finally identified 21 overlapping genes shared by the two gene sets, which were used for subsequent analyses (**Figure 1E**).

### 3.3 Functional enrichment analysis of overlapping genes

GO enrichment analysis showed that these genes were mainly enriched in biological processes including collagen fibril organization, extracellular matrix organization, and ossification. At the cellular component level, they were significantly enriched in collagen‑containing extracellular matrix, collagen trimer complexes, and fibrillar collagen trimers. For molecular function, enrichment included extracellular matrix structural constituent, platelet‑derived growth factor binding, and SMAD binding.

These processes are closely associated with environmental exposure, metabolic dysregulation, and synaptic dysfunction, which are implicated in CAG/GC-depression comorbidity (**Figure 1F**). KEGG pathway analysis further demonstrated that these genes were significantly enriched in three functional categories: neuropsychiatric‑related signaling (e.g., Notch signaling pathway, GABAergic synapse, serotonergic synapse), extracellular matrix‑adhesion signaling (e.g., ECM‑receptor interaction, focal adhesion, integrin signaling), and tumor‑associated signaling (e.g., PI3K‑Akt signaling pathway), highlighting their multifaceted roles in CAG/GC‑depression comorbidity (**Figure 1G**).

### 3.4 SOX2 is characterized as a candidate core gene correlated with disease progression

**Figure 2:**
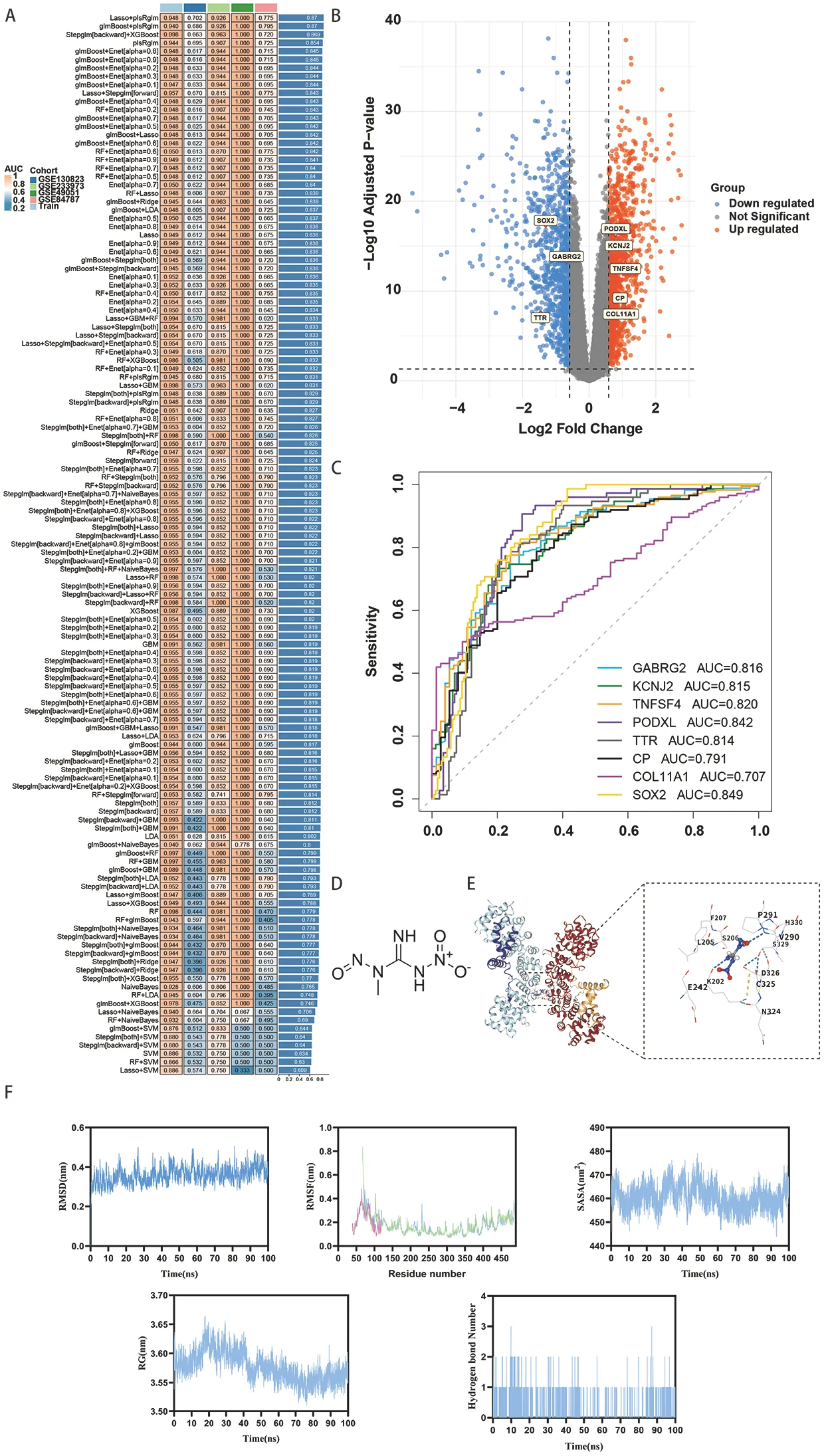
Bioinformatic identification and in silico validation of SOX2 in gastric carcinogenesis. **(A)** Model Performance Comparison: heatmap shows AUC values for various models across cohorts. Left column = models, right column = AUC (higher = better). Colors indicate cohort sources (GSE130823, GSE233973, GSE49051, and GSE84787 as external validation sets). **(B)** Volcano Plot: volcano plot shows DEGs. **(C)** ROC Curves: ROC curves for key genes. **(D)** Chemical structure of MNNG. **(E)** Docking results of SOX2 with MNNG. **(F)** Molecular Dynamics Simulation of SOX2 with MNNG.

Subsequently, machine learning analysis of the 21 candidate genes was performed to screen core diagnostic genes. The XGBoost model exhibited optimal performance with the highest accuracy in both training and validation phases (**Figure 2A**). We identified 8 pivotal genes, among which SOX2 showed the highest diagnostic value with an area under the receiver operating characteristic curve (AUC) of 0.849 (**Figures 2B, C**).

To preliminarily explore the potential interaction between the modeling carcinogen MNNG and SOX2, molecular docking and MD simulation analyses were conducted. Molecular docking results showed that MNNG could physically bind to SOX2 with a binding energy of -5.5 kcal/mol (**Figures 2D, E**). A 100 ns MD simulation was further performed to evaluate the dynamic characteristics of the MNNG–SOX2 complex. Multiple biophysical parameters, including root mean square deviation (RMSD), root mean square fluctuation (RMSF), solvent accessible surface area (SASA), radius of gyration (Rg), and intermolecular hydrogen bonds, were quantitatively analyzed (**Figure 2F**).

### 3.5 MNNG exposure coincides with arrested weight gain progression in model rats

**Figure 3:**
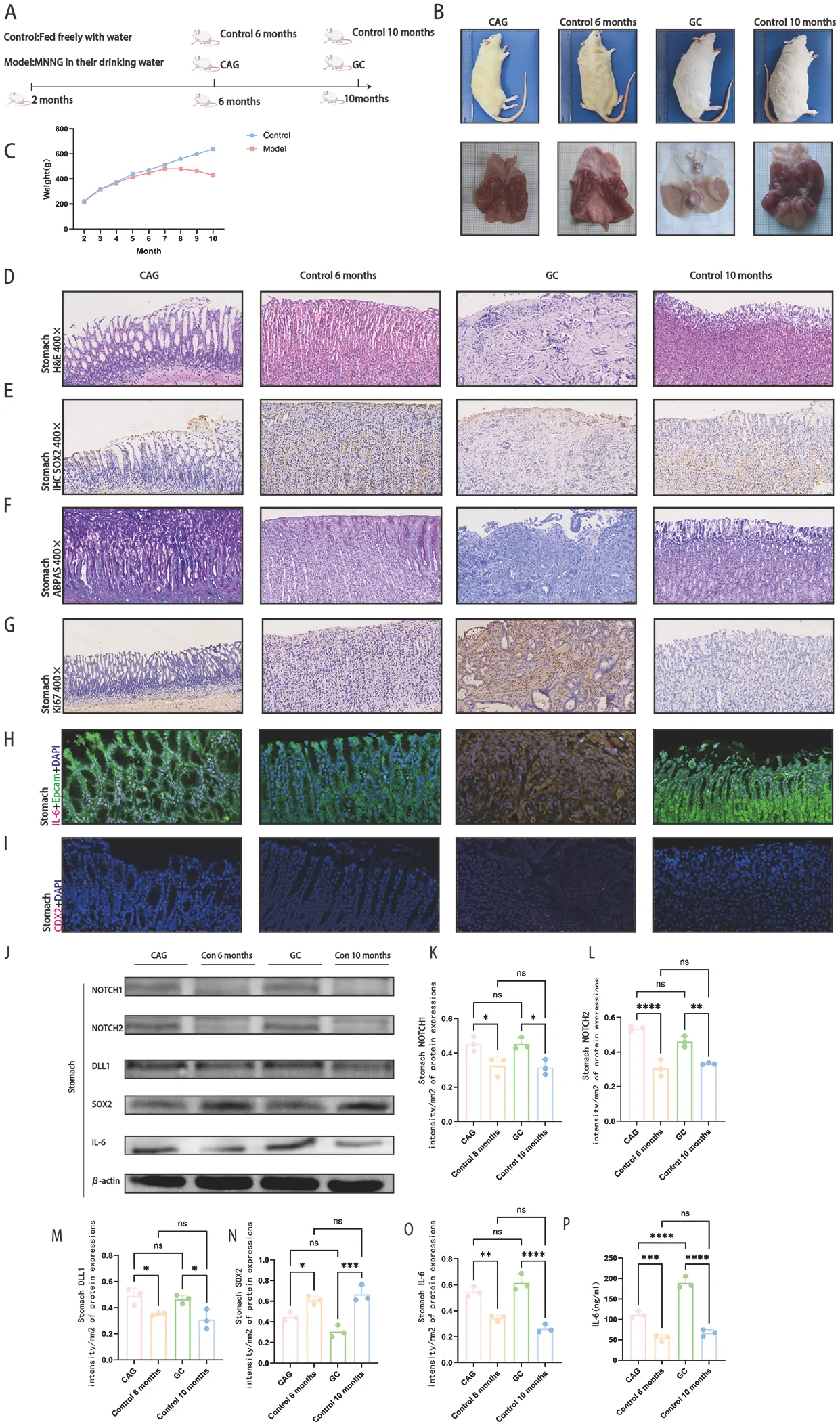
Establishment of a rat model of gastric carcinogenesis and characterization of NOTCH pathway activation and inflammatory changes in gastric tissue. **(A)** Protocol for Rat Model Establishment. **(B)** Rat Tissue Collection and Gross Observation. **(C)** Body Weight Changes. **(D)** H&E Staining of Rat Gastric Tissues. **(E)** IHC Staining of SOX2 in Rat Gastric Tissues. **(F)** AB-PAS Staining of Rat Gastric Tissues. **(G)** IHC Staining of Ki67 in Rat Gastric Tissues. **(H)** IL-6+EPCAM+DAPI Staining of Rat Gastric Tissues. **(I)** CDX2+DAPI Staining of Rat Gastric Tissues. **(J)** Expression Status of NOTCH1/NOTCH2/DLL1/SOX2/IL-6 Protein in Rat Gastric Tissues. **(K)** Quantification of NOTCH1 Protein in Rat Gastric Tissues(*F*_(3,8)_= 8.621;*P*=0.0069; η2 p =0.764). **(L)** Quantification of NOTCH2 Protein in Rat Gastric Tissues(*F*_(3,8)_= 39.42;*P*<0.0001; η2 p =0.937). **(M)** Quantification of DLL1 Protein in Rat Gastric Tissues(*F*_(3,8)_= 6.157;*P*=0.0184; η2 p = 0.697). **(N)** Quantification of SOX2 Protein in Rat Gastric Tissues(*F*_(3,8)_= 8.341;*P*=0.0076; η2 p =0.758). **(O)** Quantification of IL-6 Protein in Rat Gastric Tissues(*F*_(3,8)_= 37.660;*P*<0.0001; η2 p =0.934). **(P)** Elevated serum IL-6 levels in CAG and GC rat models(*F*_(3,8)_= 109.7;*P*<0.0001; η2 p=0.976). Statistical significance was defined as *P <0.05, **P <0.01, ***P <0.001, ****P <0.0001, and ns, not significant (*P* ≥ 0.05).

During the experimental period, the rats in the normal control group showed a steady weight gain trend with a stable and uniform growth rate; in contrast, the weight gain of the rats in the model group tended to stagnate at approximately 6 months into the experiment, without a continuous upward trend, and ultimately their body weight was significantly lower than that of the normal control group (**Figures 3A–C**).

### 3.6 SOX2 downregulation accompanied by activated NOTCH/IL-6 signaling during CAG-to-GC progression in gastric tissue

Gross observation revealed that normal gastric mucosa was pale pink with a smooth surface and regular folds. In contrast, the CAG group exhibited mucosal thinning, reduced folds, and a granular surface with visible vascular transparency. The GC group presented with localized grayish-white or dark-red lesions, characterized by a rough, uneven surface, disrupted folds, irregular nodules, and reduced tissue elasticity (**Figure 3B**).

H&E staining of rat gastric tissues was pathologically double-blind reviewed by two professional pathologists according to WHO gastric tumor classification criteria and Correa cascade staging system. In the 6-month model group with CAG, gastric mucosal glands exhibited diffuse atrophy, decreased gland number, lamina propria inflammatory infiltration, without high-grade dysplasia or malignant transformation. For the 10-month model group with GC, all induced malignant lesions were diagnosed as poorly differentiated tubular adenocarcinoma, while mucinous adenocarcinoma and signet ring cell carcinoma were not detected. Tumor lesions displayed severe glandular structural destruction, marked cellular atypia, disordered nuclear polarity and abundant mitoses; invasive growth analysis confirmed tumor cells penetrated the gastric submucosa and invaded into the muscularis propria, and none of the lesions reached the serosa layer. No malignant pathological changes were observed in gastric tissues of age-matched 6-month and 10-month control rats, with intact, regularly arranged gastric mucosal glands. (**Figure 3D**). IHC staining revealed that SOX2 was normally expressed in the proliferative zones of the gastric gland base and neck in normal mucosa. Notably, SOX2 expression was progressively downregulated during disease progression: it was significantly reduced in CAG tissues and further silenced in GC tissues, with only a small proportion of weakly positive cells observed in carcinoma lesions (**Figure 3E**). AB-PAS staining showed normal mucus secretion in the normal group, while mucus production was gradually impaired in CAG and GC groups, with most cancer cells lacking mucus staining (**Figure 3F**). IHC staining for Ki-67 revealed sparse positive proliferative cells confined to the neck and basal zones of normal gastric glands. Ki-67 proliferative index gradually increased with disease progression: Ki-67-positive cells were markedly elevated in CAG tissues and abundantly distributed across epithelial layers in GC lesions, with extensive strong nuclear staining in tumor tissues (**Figure 3G**).

IF staining of gastric tissues further confirmed characteristic inflammatory and epithelial morphological alterations across all experimental groups. Marked co-localization of interleukin-6 and the epithelial biomarker EPCAM was observed in gastric tissues of chronic atrophic gastritis model rats, indicating prominent elevation of epithelial-derived inflammatory signals. Six-month-old age-matched normal rats exhibited faint basal IL‑6 expression and intact epithelial structures. In contrast, rats with gastric malignant lesions exhibited severe glandular structural destruction and obvious cellular atypia, accompanied by striking IL-6 overexpression; these pathological manifestations are consistent with disrupted epithelial integrity in the process of gastric tumorigenesis. Although ten-month-old age-matched normal rats retained intact epithelium, they presented mild age-related inflammatory activation. (**Figure 3H**). CDX2 and DAPI co-staining IF revealed a progressive increase in CDX2 expression along the progression of gastric lesions. No obvious CDX2 fluorescent signals were detected in normal gastric mucosa; only faint and sparse CDX2 immunoreactivity was observed in CAG tissues, while CDX2 signals were markedly elevated and reached peak intensity in GC lesions (**Figure 3I**).

Western blot analysis further confirmed the activation of NOTCH signaling and inflammatory response in gastric tissues. Compared to age-matched controls, CAG and GC groups showed significantly elevated protein levels of NOTCH1, NOTCH2, and their ligand DLL1, accompanied by upregulated pro-inflammatory cytokine IL-6. Concurrently, SOX2 expression was markedly downregulated in both CAG and GC groups (**Figures 3J–P**). Collectively, these data indicate concurrent elevation of inflammatory cytokines and NOTCH pathway activation alongside reduced SOX2 abundance in gastric tissues, revealing a correlative pattern among these molecular alterations.

### 3.7 SOX2 reduction and NOTCH/IL-6 hyperactivation in brain tissue along CAG-to-GC progression

**Figure 4:**
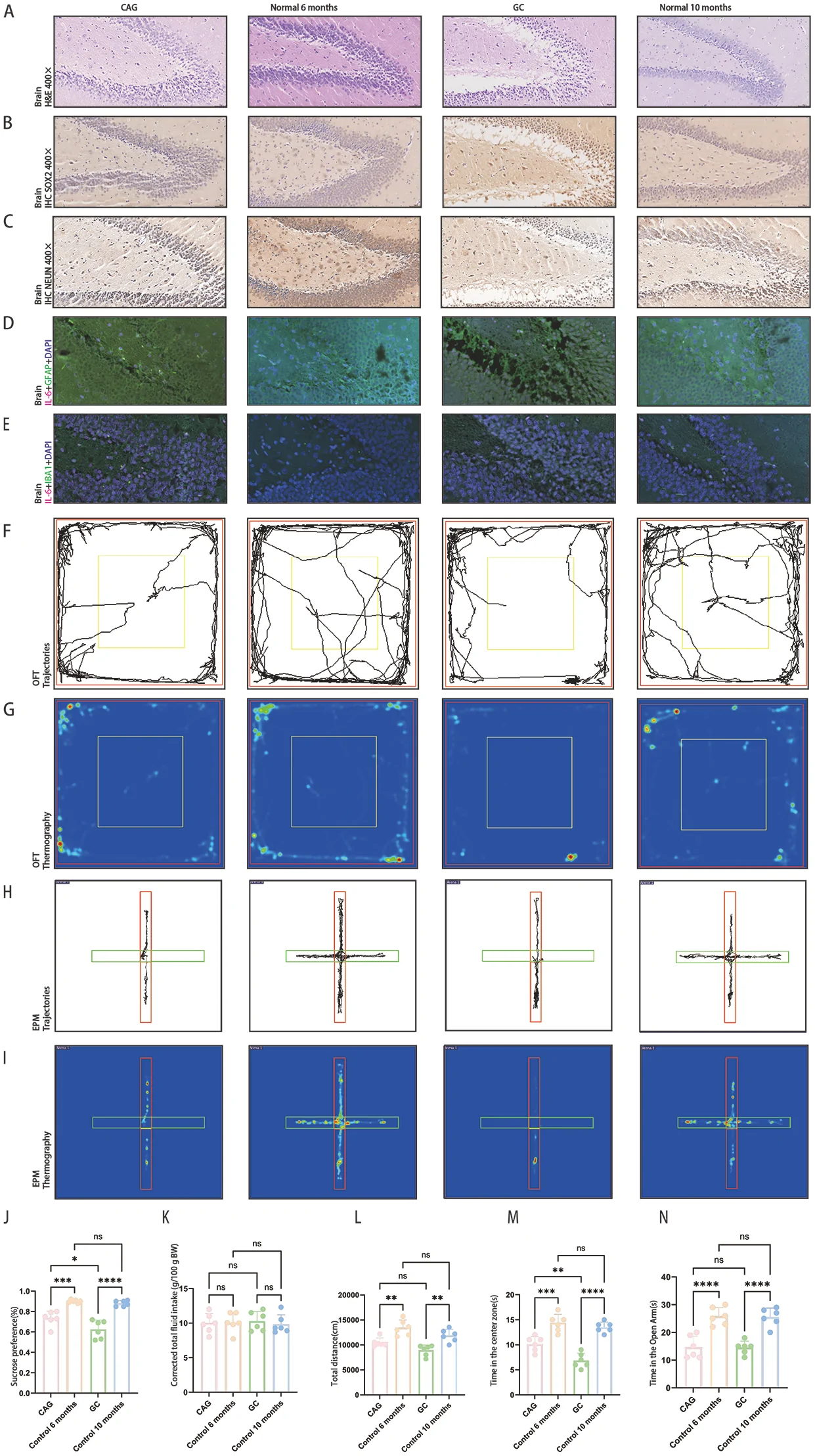
MNNG-induced CAG/GC rat models exhibited reduced activity and anhedonia-like behavior, along with concurrent neurohistological alterations. **(A)** H&E Staining Status of the DG Region in Rat Hippocampal Tissues. **(B)** IHC Staining Status of SOX2 in the DG Region of the Hippocampus in Rat. **(C)**IHC Staining Status of NEUN in the DG Region of the Hippocampus in Rat. **(D)** IL-6+GFAP+DAPI Staining of the Hippocampus in Rat. **(E)**IL-6+IBA1+DAPI Staining of the Hippocampus in Rat. **(F, G)** Behavioral Experiment: Open Field Test. **(H, I)** Behavioral Experiment: Elevated plus maze(Red for closed arms, green for open arms). **(J)** Behavioral Experiment: Sucrose Preference Test(*F*_(3,20)_=28.66;*P*<0.0001; η2 p=0.811). **(K)** Corrected total fluid intake (*F*_(3,20)_=0.182;*P*=0.907;η2 p=0.017). **(L)** Total distance traveled in the OFT(*F*_(3,20)_=15.09;*P*<0.0001;η2 p=0.694). **(M)** Time spent exploring the center area in the OFT(*F*_(3,20)_=33.17;*P*<0.0001;η2 p=0.832). **(N)** Time in the Open Arm(s) (*F*_(3,20)_=23.06;*P*<0.0001;η2 p=0.776). Statistical significance was defined as *P <0.05, **P <0.01, ***P <0.001, ****P <0.0001, and ns, not significant (*P* ≥ 0.05).

Histological and immunostaining analyses of brain tissues revealed group-specific pathological alterations. H&E staining demonstrated mild structural disorganization in rats with CAG, whereas intact tissue architecture was maintained in six-month-old age-matched control rats. In contrast, brain tissue integrity was severely compromised in gastric cancer model rats, while only subtle age-related morphological changes were detected in ten-month-old age-matched normal rats. (**Figure 4A**). IHC staining showed that SOX2 expression was significantly downregulated in CAG and GC rats compared to normal controls, consistent with the changes observed in gastric tissue. Neuronal marker NEUN was reduced in GC rats, indicating neuronal loss during disease progression. Double IF staining revealed robust co-localization of IL-6 with glial markers GFAP and IBA1 in CAG and GC rats, reflecting marked neuroinflammatory and glial activation (**Figures 4B–E**).

**Figure 5:**
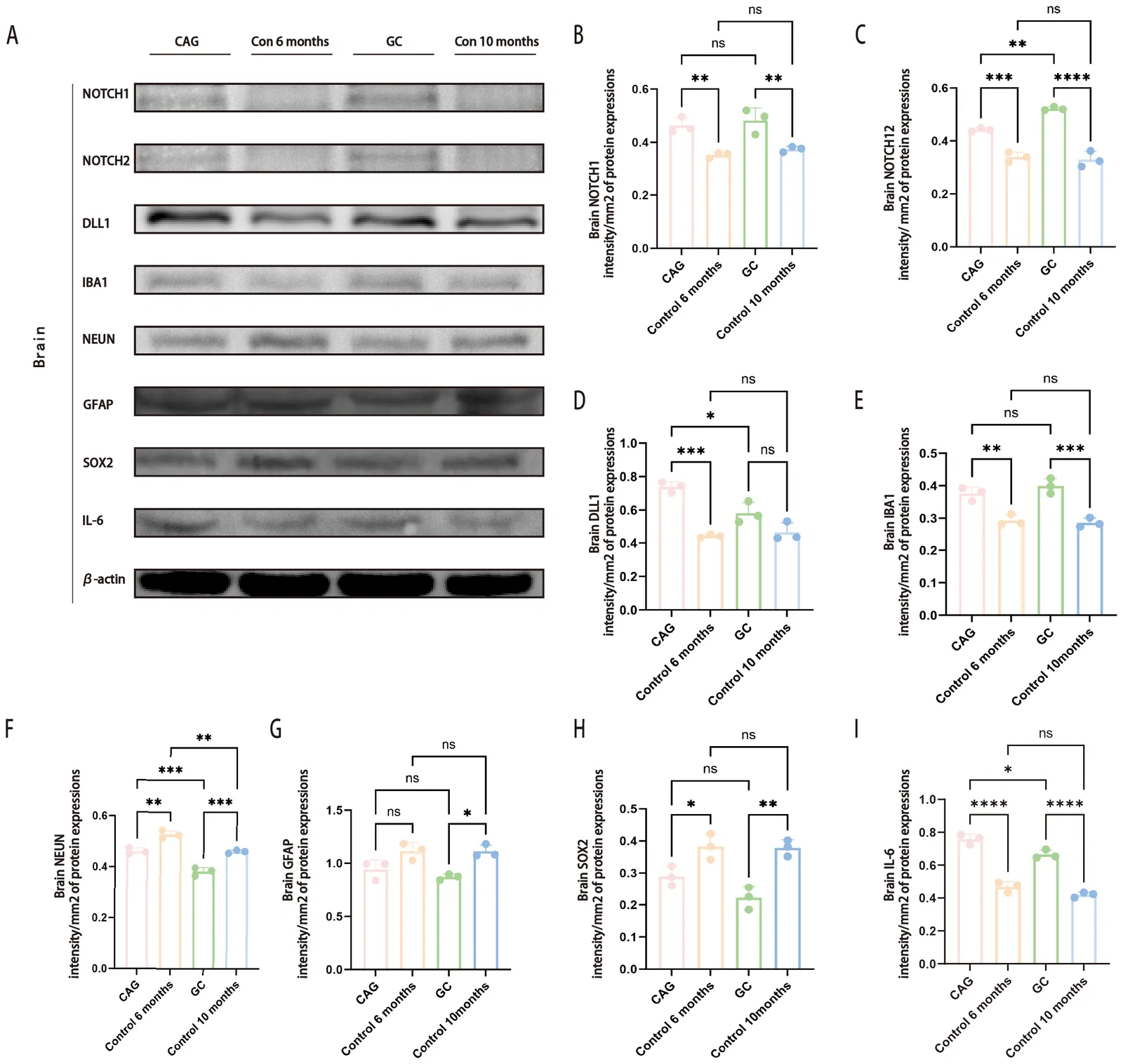
Activation of NOTCH signaling pathway, neuroinflammation, and downregulation of SOX2 in rat brain tissues. **(A)** Expression Status of NOTCH1/ NOTCH2/DLL1/ IBA1/NEUN/GFAP/SOX2/IL-6 Protein in Rat Brain Tissues. **(B)** Expression Status of NOTCH1 Protein in Rat Brain Tissues(*F*_(3,8)_= 14.91;*P*=0.0012; η2 p=0.848). **(C)** Expression Status of NOTCH2 Protein in Rat Brain Tissues(*F*_(3,8)_= 73.93;*P*<0.0001; η2 p=0.965). **(D)** Expression Status of DLL1 Protein in Rat Brain Tissues(*F*_(3,8)_= 26.28;*P*=0.0002; η2 p=0.908). **(E)** Expression Status of IBA1 Protein in Rat Brain Tissues(*F*_(3,8)_= 26.89;*P*=0.0002; η2 p=0.911). **(F)** Expression Status of NEUN Protein in Rat Brain Tissues(*F*_(3,8)_= 62.63;*P*<0.0001; η2 p=0.959). **(G)** Expression Status of GFAP Protein in Rat Brain Tissues(*F*_(3,8)_= 9.619;*P*=0.005; η2 p=0.783). **(H)** Expression Status of SOX2 Protein in Rat Brain Tissues(*F*_(3,8)_= 16.17; *P*=0.0009; η2 p=0.858). **(I)** Expression Status of IL-6 Protein in Rat Brain Tissues (F(3,8)= 46.81; *P*<0.0001; n2 p=0.9454. Statistical significance was defined as *P <0.05, **P <0.01, ***P <0.001, ****P <0.0001, and ns, not significant (*P* ≥ 0.05).

Behavioral testing was conducted to assess locomotor/exploratory activity (OFT), anhedonic-like phenotypes (SPT), and anxiety-related avoidance traits (EPM) in rats. Relative to age-matched controls, both the CAG and GC groups exhibited significantly reduced locomotor activity, decreased time spent in the central zone of the OFT, and lower sucrose preference, indicating blunted motivational and hedonic responses. In the EPM, these two groups showed diminished open-arm exploration (green frame) and increased time spent in the closed arms (red frame), consistent with elevated anxiety-like traits. Collectively, these findings reveal a consistent pattern of motor suppression, anhedonia, and heightened anxiety in both treatment groups. (**Figures 4F–N**). Western blot analysis revealed that, consistent with the findings in gastric tissue, NOTCH signaling components (NOTCH1, NOTCH2, DLL1) and IL-6 were significantly upregulated in the brain tissues of CAG and GC rats, accompanied by increased microglial activation marker IBA1. Concurrently, SOX2 expression was markedly downregulated in both tissues (**Figures 5A–H**). Collectively, these findings demonstrate that CAG and GC rats recapitulated diminished locomotor activity and anhedonic-like phenotypes, accompanied by NOTCH pathway activation, IL-6-mediated inflammation, and SOX2 downregulation in both gastric and brain tissues.

### 3.8 Activation of the NOTCH signaling pathway and upregulation of IL-6 in gastric epithelial cell lines

**Figure 6:**
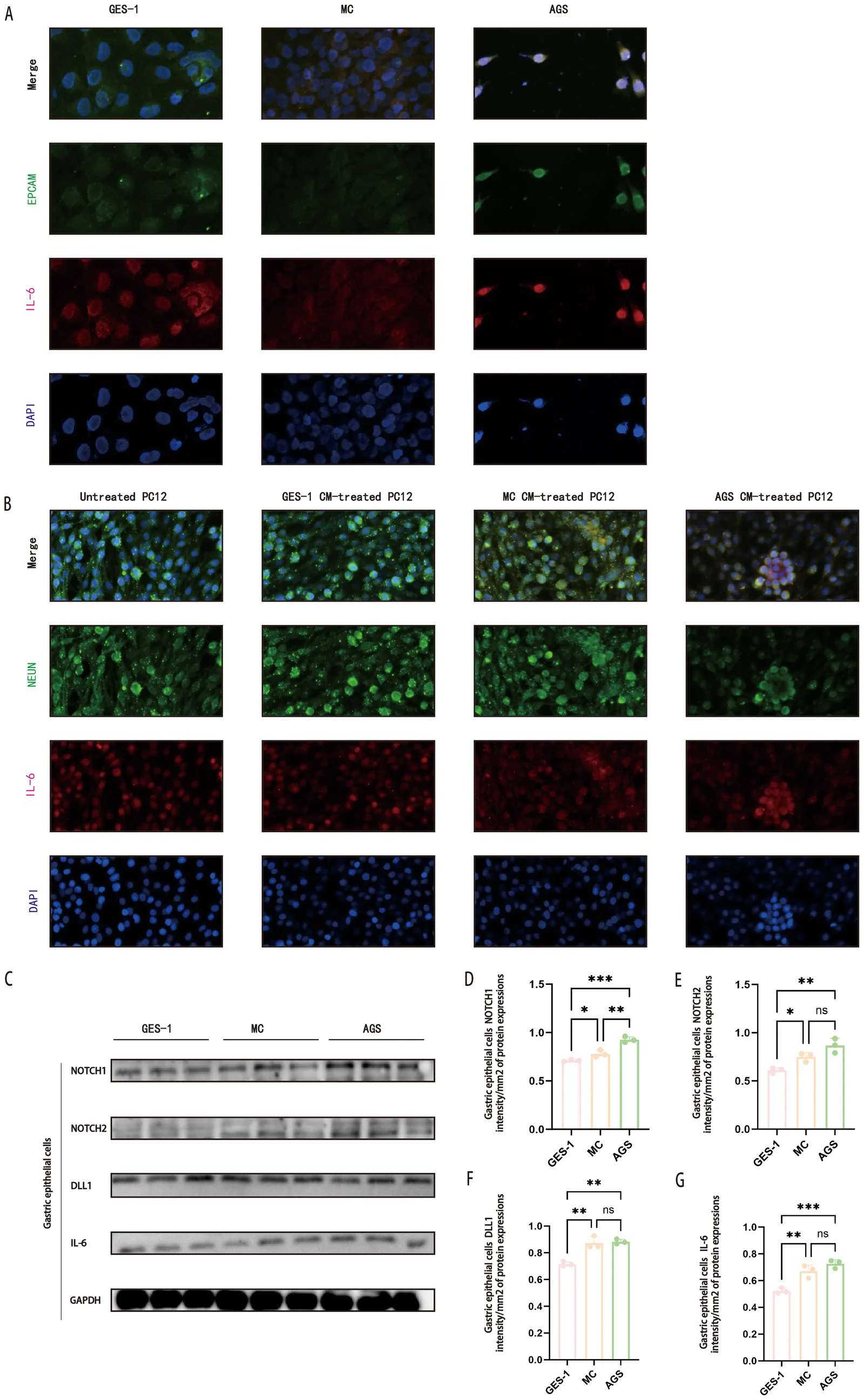
Activation of the NOTCH signaling pathway and upregulation of IL-6 in gastric epithelial cell lines. **(A)** IF of EPCAM and IL-6 in gastric epithelial cells. **(B)** IF of NEUN and IL-6 in CM-treated PC12 cells. **(C)** Western blot of NOTCH pathway molecules and IL-6. **(D)** Expression Status of NOTCH1 Protein in Gastric Epithelial Cell Lines(*F*_(2,6)_= 47.16;*P*=0.0002; η2 p=0.94). **(E)** Expression Status of NOTCH2 Protein in Gastric Epithelial Cell Lines(*F*_(2,6)_= 17.36;*P*=0.003; η2 p=0.8527). **(F)** Expression Status of DLL1 Protein in Gastric Epithelial Cell Lines(*F*_(2,6)_= 28.09;*P*=0.0009; η2 p=0.904). **(G)** Expression Status of IL-6 Protein in Gastric Epithelial Cell Lines(*F*_(2,6)_= 27.19;*P*=0.001; η2 p=0.901). Statistical significance was defined as *P <0.05, **P <0.01, ***P <0.001, and ns, not significant (*P* ≥ 0.05).

To further verify the paracrine mechanism underlying the gastric-brain crosstalk, we examined the expression of NOTCH signaling components and IL-6 in gastric epithelial cell lines. Compared to normal gastric epithelial cells (MC and GES-1), gastric cancer AGS cells showed significantly upregulated IL-6 expression, along with elevated protein levels of NOTCH1, NOTCH2, and DLL1 (**Figures 6A, C**-G). We then treated neuronal PC12 cells with conditioned medium (CM) from gastric epithelial cells to evaluate the paracrine effect. Notably, IL-6 expression was significantly upregulated in PC12 cells treated with AGS-derived CM, indicating that GC cells could induce inflammatory activation in neuronal cells via paracrine signaling (**Figure 6B**).

## 4 Discussion

CAG and GC frequently co-occur with depression in clinical practice. This comorbidity correlates with faster disease progression and poorer patient outcomes and may sustain a self-reinforcing correlative cycle. The molecular regulators linking these two disorders remain poorly characterized. This study combined transcriptomic bioinformatics and machine learning analyses to screen SOX2 as a potential shared regulatory gene correlated with GC and depression. We further validated this putative link using MNNG-induced rat models and *in vitro* cellular assays. Our preliminary observations support an association between SOX2 downregulation and bidirectional stomach–brain crosstalk in CAG/GC-depression comorbidity. Lower gastric SOX2 levels coincide with NOTCH pathway activation and enhanced IL-6-driven mucosal inflammation, alongside higher systemic IL-6 concentrations. These peripheral molecular shifts correlate with hippocampal neuroinflammation, impaired exploratory locomotion, and anhedonic-like phenotypes. Separately, reduced hippocampal SOX2 expression aligns with exacerbated central inflammatory activation, collectively forming a self-reinforcing correlative loop between gastric pathological injury and depressive-like behavioral alterations. These preliminary findings provide a potential theoretical reference and a candidate therapeutic target for future clinical research on GC-depression comorbidity.

The present study highlights the potential regulatory implication of SOX2 in GC-depression comorbidity. SOX2 is a canonical stem cell transcription factor that is essential for the maintenance of gastric mucosal epithelial homeostasis. Multiple studies have demonstrated that SOX2 hypermethylation and transcriptional silencing occur in the early stage of gastric tumorigenesis, which is associated with impaired gastric epithelial differentiation, excessive cell proliferation, and the progression from CAG to GC. Accumulating clinical and basic evidence also suggests that SOX2 dysfunction is correlated with multiple neuropsychiatric disorders. SOX2 activity is closely related to hippocampal neurogenesis, and reduced SOX2 expression is associated with defective neuronal differentiation and neuroinflammation, which are potentially linked to the pathogenesis of depression. Relevant investigations focusing on the regulatory role of SOX2 in gastric disease-depression comorbidity are still limited. In the present study, synchronous downregulation of SOX2 in both gastric and hippocampal tissues was observed in CAG/GC rats with depressive-like behaviors. Molecular docking and MD simulation analyses suggest stable binding affinity between the gastric carcinogen MNNG and SOX2, which may partially explain the reduced SOX2 abundance in peripheral gastric and central hippocampal tissues. Collectively, these preliminary results imply that SOX2 dysregulation may be a potential linking factor for the comorbidity of gastric diseases and depression.

Our data further suggest that the NOTCH/IL-6 inflammatory pathway may serve as a downstream regulatory network by which SOX2 correlates with stomach–brain crosstalk. NOTCH signaling is a conserved pathway regulating inflammation, cell fate determination, and tissue homeostasis. Activated NOTCH signaling is reportedly involved in gastric mucosal inflammatory injury and CAG/GC progression, and also contributes to hippocampal microglial activation and neuroinflammation, which are closely associated with depressive-like behaviors. As a key pro-inflammatory cytokine downstream of NOTCH signaling, IL-6 acts as a critical messenger in stomach–brain communication. In our study, SOX2 downregulation was accompanied by NOTCH pathway activation and elevated IL-6 levels in both gastric and hippocampal tissues. In addition, SOX2 overexpression could partially reverse NOTCH/IL-6 pathway activation in GC cells. These observations support a negative correlation between SOX2 and the NOTCH/IL-6 inflammatory axis, which may represent a plausible mechanism underlying SOX2-associated GC-depression comorbidity.

More importantly, our study verified the key link of “peripheral inflammation entering the circulation to induce central neuroinflammation” in the stomach-brain axis, which is the core of our research hypothesis. We found that serum IL-6 levels were significantly elevated in CAG/GC rats, and were positively correlated with the severity of reduced activity and anhedonia-like behavior. *In vitro* experiments further confirmed that GC cell-derived conditioned medium was accompanied by IL-6 inflammatory activation in neuronal cells. These findings support an association between gastric epithelial-derived IL-6 elevation and central inflammatory changes, but do not constitute direct causal evidence that circulating IL-6 enters the brain to trigger neuroinflammation *in vivo*. Consistent with our findings, previous studies have shown that peripheral IL-6 can cross the blood-brain barrier, activate microglia and astrocytes in the hippocampus, induce neuroinflammation, and ultimately lead to reduced activity and anhedonia-like behavior. Our work links this inflammatory cascade with SOX2 dysfunction and proposes a putative mechanistic sequence: SOX2 downregulation correlates with gastric NOTCH/IL-6 pathway activation, which raises circulating IL-6 concentrations, then correlates with hippocampal neuroinflammation, and eventually associates with diminished locomotor activity and anhedonic-like phenotypes.

Several notable limitations exist in the present study. First, although we observed a regulatory correlation between SOX2 and the NOTCH/IL-6 signaling axis at both cellular and animal levels, our current data are correlative rather than causal; the precise direct molecular mechanism mediating this crosstalk remains unknown, and definitive mechanistic experiments—such as conditional knockout, rescue assays, or pathway-specific inhibitors—are required to establish causality. Second, high-quality, uniformly standardized CAG transcriptomic datasets with rigorous clinical grouping remain relatively limited in public databases. In the present study, we supplemented and integrated two eligible CAG datasets alongside qualified GC datasets for preliminary screening, which substantially improved the coverage of preneoplastic gastric lesion transcriptomic information. Nevertheless, the currently available CAG datasets still exhibit relatively small sample sizes and cross-platform heterogeneity, which may introduce potential batch effects and partially limit the robustness of preneoplastic gene screening. To further optimize and refine the regulatory network of CAG/GC-depression comorbidity, future studies will integrate larger-scale, multi-platform CAG transcriptomic data to systematically mine preneoplastic-specific core genes and extend the current research framework. Third, IL-6 is characterized as a core regulator of stomach–brain axis communication in progressive lesions in our research. However, additional inflammatory mediators, neurotransmitters and neuroendocrine signaling cascades could jointly drive this comorbid pathology. Subsequent behavioral tests such as the forced swimming test should be performed to comprehensively clarify their biological functions underlying stomach–brain axis impairment. In addition, our molecular docking and MD simulations suggest a possible direct physical interaction between MNNG and SOX2, as reflected by a binding energy of −5.5 kcal/mol and complex stability over 100 ns. However, these in silico findings are preliminary and do not support an association linking MNNG exposure to suppressed SOX2 expression or impaired protein function. Confirmatory biochemical and genetic experiments, such as surface plasmon resonance, cellular thermal shift assay, and site-directed mutagenesis, are needed to verify this putative interaction and assess its functional impact.

In conclusion, this study suggests that SOX2 may serve as a potential core mediator correlated with the bidirectional crosstalk between CAG/GC and depression, possibly through modulating the NOTCH/IL-6 inflammatory pathway of the stomach–brain axis. Our findings provide preliminary insights into the potential molecular correlation of GC–depression comorbidity, which may offer promising candidate biomarker and therapeutic target for future clinical investigations of this comorbid condition.

## Data Availability

The datasets presented in this study can be found in online repositories. The names of the repository/repositories and accession number(s) can be found below: https://www.ncbi.nlm.nih.gov/, GSE118916, GSE54129, GSE65801, GSE60427 GSE130823,GSE233973, GSE49051, and GSE84787.
